# Insulin sensitivity in individuals with burnout is associated with physical activity level – a study using oral glucose tolerance test

**DOI:** 10.3389/fpsyt.2026.1664680

**Published:** 2026-02-05

**Authors:** Anna-Karin Lennartsson, Ingibjörg H. Jonsdottir, Per-Anders Jansson, Anna Sjörs Dahlman

**Affiliations:** 1 The Institute of Stress Medicine, Region Västra Götaland, Gothenburg, Sweden; 2 School of Public Health and Community Medicine, Institute of Medicine, Sahlgrenska Academy at the University of Gothenburg, Gothenburg, Sweden; 3 Wallenberg Laboratory, Department of Molecular and Clinical Medicine, Institute of Medicine, Sahlgrenska Academy, University of Gothenburg, Gothenburg, Sweden; 4 Swedish National Road and Transport Research Institute, Linköping, Sweden; 5 Department of Electrical Engineering, and SAFER Vehicle and Traffic Safety Centre, Chalmers University of Technology, Gothenburg, Sweden

**Keywords:** burnout, HOMA-IR, insulin sensitivity, Matsuda index, oral glucose tolerance test, physical activity

## Abstract

**Aims:**

Burnout is caused by long term psychosocial stress and has, besides the fatigue and mental health burden, been associated with increased risk of adverse physical health, such as type 2 diabetes. Physical activity seems to be a protective factor against burnout and its negative health consequences. This study aims to investigate the glucose and insulin levels related to physical activity level in individuals with stress related burnout, by assessing these metabolic markers in response to a standard oral glucose tolerance test (OGTT).

**Methods:**

Altogether, 38 individuals with burnout (13 men and 25 women) in the age 24–55 were included in the study. The burnout cases were divided into three groups based on self-reported physical activity level.

**Results:**

The burnout cases who reported that they were sedentary exhibited significantly higher insulin levels during the OGTT compared to burnout cases reporting that they were physically active to any degree. This relationship was independent of severity of symptoms of burnout and depression.

**Conclusions:**

The observed higher insulin levels in the sedentary burnout cases indicate an increased diabetes risk in these individuals and point at an important reason for physical activity being included in the treatment regimen for this patient group.

## 1 Introduction

Burnout is a well-described consequence of long-term stress, with exhaustion being one of its core components (1). Besides fatigue, burnout is often accompanied by symptoms of depression and anxiety. Furthermore, burnout is associated with increased risk of adverse physical health (1–7), including increased risk of developing type 2 diabetes (5, 8, 9). Prospective studies have shown that burnout has a two- to threefold increase in risk of developing type 2 diabetes (5, 9). The suggested mechanism behind this association is that psychosocial stress, via neuroendocrine pathways, induces insulin resistance (10–12), a pre-diabetic state which implicates reduced capacity to transport glucose into cells and thus reduced capacity to utilize glucose as an energy source. Thus, peripheral tissues such as skeletal muscle, liver and adipose tissue do not respond sufficiently to physiological insulin concentrations. To compensate for this, the beta cells of the pancreas produce more insulin, which, if not sustained, could eventually result in chronic hyperglycemia and type 2 diabetes (13). We have previously investigated insulin sensitivity and glucose control in 38 individuals with burnout, using an oral glucose tolerance test (OGTT), and found that those with more severe burnout symptoms exhibited significantly higher levels of both glucose and insulin levels during the OGTT compared to those who reported lower severity of symptoms. In addition, the group of burnout cases who reported symptoms of depression exhibited higher insulin levels during OGTT compared to those without depressive symptoms. In the present article we investigate, in the same participants, the importance of physical activity for insulin sensitivity. Physical activity is known to influence insulin sensitivity and glucose control beneficially, both in patients with type 2 diabetes (14) and in healthy individuals (15). It could, however, be speculated that the relation between physical activity and glucose control may be disturbed in patients with burnout due to the extensive stress exposure affecting different neuroendocrine systems. Thus, chronic stress can disrupt the neuroendocrine regulation of glucose metabolism (8, 16), and this may interfere with the expected benefits of physical activity on glucose control. To our knowledge, the relation between physical activity level and glucose control has not been previously studied in burnout cases. Thus, in the present study, we aim to investigate whether glucose and insulin levels, and measures of insulin sensitivity, in individuals experiencing burnout, are related to physical activity level using oral glucose tolerance test.

## 2 Method

### 2.1 Participants

Thirty-eight individuals with burnout (13 men and 25 women) in the age 24–55 were included in the study. The participants were recruited via advertisements in waiting rooms at occupational health care and primary care units in Gothenburg, Sweden. Inclusion criteria for burnout cases were a mean score ≥4.4 on the Shirom-Melamed Burnout Questionnaire (SMBQ) and self-rated symptoms for severe exhaustion disorder assessed with the self-rated exhaustion disorder (s-ED) questionnaire. A mean SMBQ score above 4.4 has previously been shown to discriminate patients with clinical burnout from healthy controls (17). The s-ED questionnaire is based on the Swedish diagnostic criteria for Exhaustion Disorder, which is the clinical diagnosis used for burnout in Sweden (18). Before inclusion, the subjects underwent a screening test, including anthropometric measurements. Blood samples were obtained. The screening was performed to assess the following exclusion criteria: having a body mass index less than 18.5kg/m^2^ or higher than 30kg/m^2^, hypertension (blood pressure>140/90 mmHg), infection, anemia, vitamin B12 deficiency (high homocysteine), known systemic disease such as diabetes or thyroid disease, known psychiatric disease (except self-rated stress-related exhaustion, depression and anxiety), alcohol abuse, pregnancy, breast feeding, menopause or use of drugs with systemic effects, including oral contraceptives but excluding antidepressants for the burnout cases. All participants gave written informed consent before entering the study and were informed that they could withdraw their participation at any time. The study was conducted according to the Declaration of Helsinki and approved by the Regional Ethical Board, Gothenburg, Sweden, Dnr 755-15.

### 2.2 Oral glucose tolerance test

The participants underwent an OGTT performed according to the World Health Organization (WHO) criteria of 1985. While a single glucose measurement can only define impaired fasting glucose (iFG), OGTT mirrors the patient’s ability to normalize the glucose levels after glucose intake, thus it detects impaired glucose tolerance (iGt). Furthermore, OGTT offers the opportunity to study insulin resistance and beta-cell function, by HOMA indexes, using fasting levels of glucose and insulin. A venous catheter was inserted to enable blood sampling at regular intervals. During the test, study participants rested sitting in an armchair reading or listening to the radio. Directly before the glucose load, the first blood samples were taken, constituting the fasting samples. The subjects then received 200 ml of glucose dissolved in water (75 g of glucose) and blood samples were drawn 30, 60, 90 and 120 minutes after the glucose load. Total blood loss was about 110 ml. Blood samples for analysis of plasma glucose and serum insulin were sent directly to the Laboratory for Clinical Chemistry at Sahlgrenska University Hospital for analysis.

### 2.3 Glucose and insulin measures

The standard American Diabetes Association (ADA) criteria were used for the diagnosis of diabetes and pre-diabetes. 2-hour venous plasma glucose value ≥ 11.1 mmol/L was classified as diabetes and 7.8-11.0 mmol/L was classified as impaired glucose tolerance (IGT). HOMA-IR (Homeostatic Model Assessment for Insulin Resistance) (19) and the Matsuda Index (20), indicators of insulin resistance and insulin sensitivity, were calculated (fasting glucose x fasting insulin)/22.5 and 10000/√(fasting glucose x fasting insulin x mean glucose during OGTT x mean insulin during OGTT), respectively. Area under the curve (AUC) for glucose levels during the OGTT was calculated, with the formula; AUC(mmol/L*min) = 1/2 ×(PG 0 min+PG 30 min)×30 min +1/2×(PG 30 min+PG 60 min)×30 min +1/2×(PG 60 min+PG 90 min)×30 min +1/2×(PG 90 min+PG 120 min)×30 min. AUC for insulin was calculated in correspondingly.

### 2.4 Questionnaires

The participants answered several questionnaires. Besides assessing symptoms of burnout, anxiety, and depression, they also answered questionnaires regarding their physical activity level. The Shirom-Melamed Burnout Questionnaire (SMBQ) (1) was used to measure severity of burnout symptoms. SMBQ contains 22 items (graded 1-7) measuring the different aspects of burnout: emotional and physical exhaustion, tension, listlessness, and cognitive weariness. A mean burnout score was calculated for each participant. The Hospital Anxiety and Depression (HAD) scale was used to assess self-reported symptoms of depression and anxiety (21, 22). HAD contains 14 items (7 items for each subscale). The scores for each subscale were used to classify “non-cases” (0-7), “possible cases” (8-10), and “cases” (above 10) of anxiety and depression, respectively. Participants reported their physical activity level (the past year) with the Saltin-Grimby Physical Activity Level Scale (23), which has been shown to be a useful screening measure of physical activity (24). This scale is a single-item question with four response options. The first level corresponds to a sedentary lifestyle, while levels 2–4 represent activity levels from light to strenuous exercise training.

### 2.5 Statistical analysis

Kolmogorov-Smirnov test was used for each study variable to test whether data were normally distributed. The variables which showed a non-normal distribution underwent logarithmic transformation. After logarithmic transformation (ln), the Kolmogorov-Smirnov test was used again to control whether the new variable showed a normal distribution.

Of the 38 individuals with burnout, 11 reported that they were sedentary, 13 reported some light physical activity, 13 reported regular physical activity/training and one reported regular hard physical training for competitive sports. The single participant who reported hard physical training was merged with the group who reported regular physical activity/training. Thus, three physical activity groups were formed; Sedentary (n=11), Light physical activity (n=13) and Moderate to hard physical activity (n=14). Background variables were compared between these physical activity groups. Two-way ANOVA was used analyzing possible differences in age, BMI, and WHR; Kruskal-Wallis H test was used for burnout score, depression score, and anxiety score, and Chi-Square test for proportion of males/females, tobacco use and use of antidepressants. Figures were created and these showed that the two groups who were physically active exhibited very similar values all throughout. Further statistical comparisons were therefore performed between two groups; those who were sedentary and those who were active (response option 1 vs. 2–4 on the Saltin-Grimby Physical Activity Level Scale). The distribution of men and women in the two groups was compared using the Chi-square test. T-tests were used to compare age and BMI between the groups. The Mann-Whitney U test was used to compare scores of burnout and depression between sedentary and physically active burnout cases. Levels of HbA1c and fasting glucose (measured at screening) were compared between sedentary and active participants using t-tests. To investigate possible differences in response to OGTT between sedentary and physically active burnout cases, mixed between and within analysis of variance (ANOVA) including interaction was computed with hormonal level (insulin and glucose, respectively) at the five different time points as the within variable and Group (Sedentary vs Active) as the between variable. Log values of glucose and insulin were used. AUC glucose (log), AUC insulin (log), the Matsuda Index and HOMA-IR (log) were compared between sedentary and active subjects using t-tests. Regression analyses were performed to investigate whether the observed effect of physical activity (Sedentary vs. Active) on insulin sensitivity (AUC insulin, HOMA-IR, and Matsuda Index) persisted after adjusting for age, BMI, gender.

## 3 Results

**Table 1** reports background characteristics in the three groups with different physical activity levels.

**Table 1:** Background characteristics in the 38 participants; burnout cases with different levels of self-reported physical activity.

Characteristics	Sedentary n=11	Light physical activity n=13	Moderate physical activity n=14	p value
	Mean (±SD)*	Mean (±SD)*	Mean (±SD)*	
Age, years	39 (8)	43 (8)	44 (8)	0.289
Sex (% women)	64%	69%	64%	0.949
BMI, kg/m²	23.2 (3.2)	22.5 (3.0)	22.2 (3.7)	0.737
WHR	0.85 (0.08)	0.81 (0.07)	0.82 (0.07)	0.406
Tobacco use (snuffing or smoking)	9%	0%	14%	0.382
Antidepressant use	36%	23%	29%	0.774
Burnout score (SMBQ)	5.67 (4.28-6.83)	5.72 (4.00-6.67)	5.19 (4.33-6.33)	0.392
Depression score (HAD)	7.00 (4.00-14.0)	7.00 (2.00-17.0)	7.50 (2.00-17.0)	0.551
Anxiety score (HAD)	9.00 (1.00-15.0)	12.0 (6.00-18.0)	10.0 (5.00-17.0)	0.247

BMI, Body Mass Index; WHR, Waist Hip Ratio; HAD, Hospital Anxiety Depression Scale; SMBQ, Shirom-Melamed Burnout Questionnaire. Statistical comparison between the groups were done using two-way ANOVA for age, BMI and WHR, Kruska-Wallis H test for Burnout score, Depression score, and Anxiety score, and Chi-Square test for tobacco use and use of antidepressants. *For Burnout score, Depression score and Anxiety score Median (range) are reported instead of Mean (± SD).

**Figure 1:**
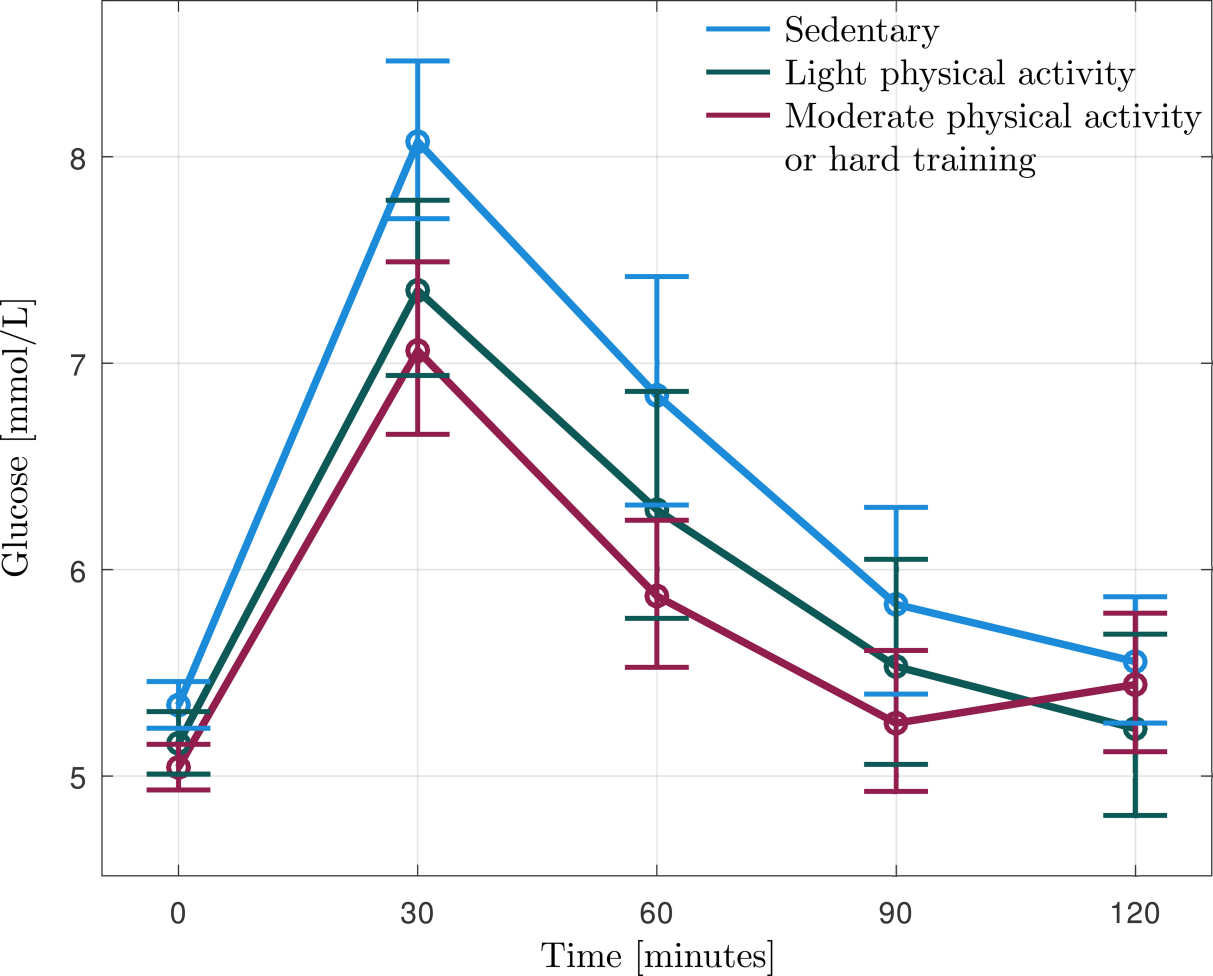
Geometric mean (95% CI) of glucose levels during oral glucose tolerance test (OGTT) in the burnout subjects reporting that they are sedentary (n=11), practice some light physical activity (n=13), or regular physical activity (n=14).

**Figure 2:**
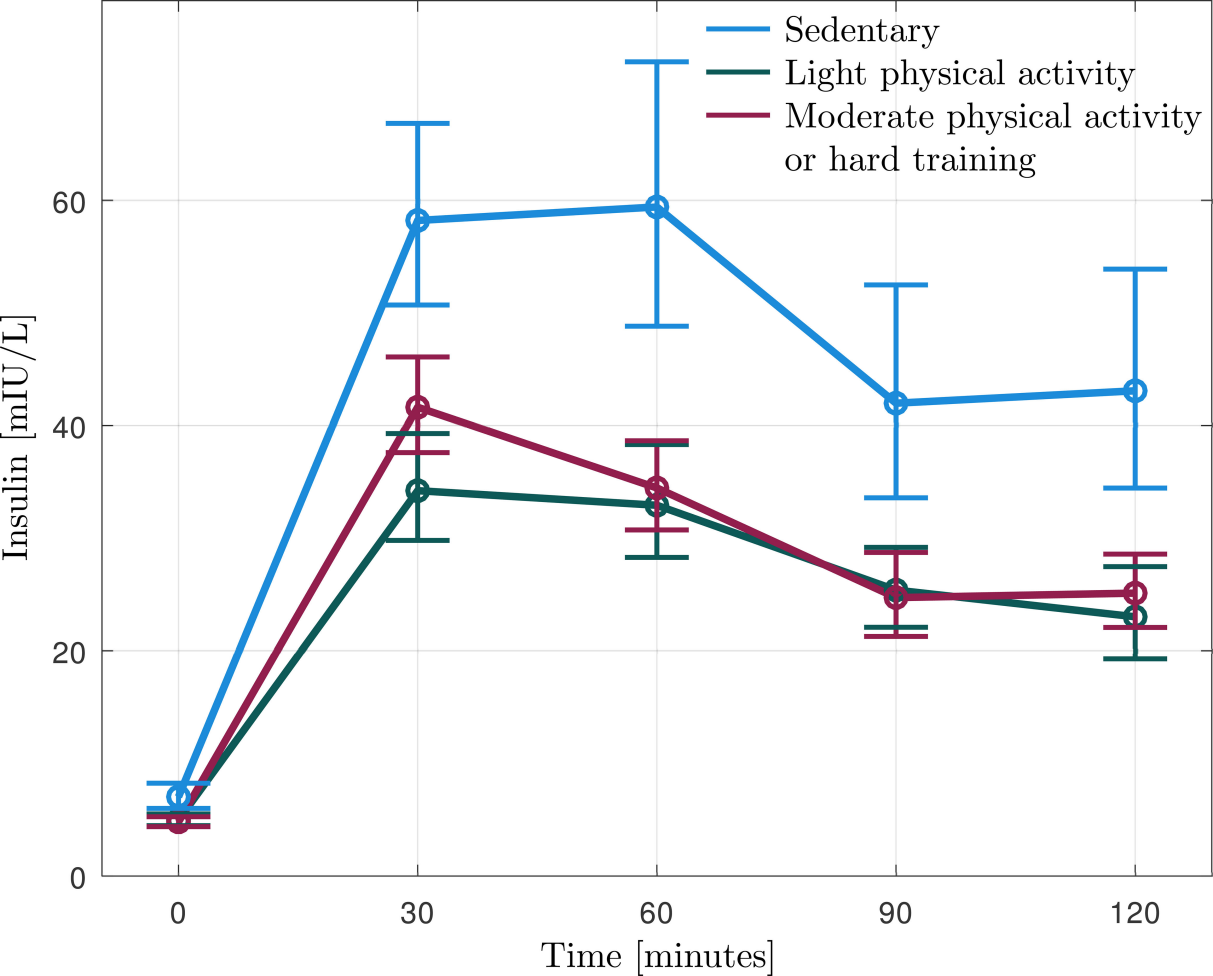
Geometric mean (95% CI) of insulin levels during oral glucose tolerance test (OGTT) in the burnout subjects reporting that they are sedentary (n=11), practice some light physical activity (n=13), or regular physical activity (n=14).

**Figure 3:**
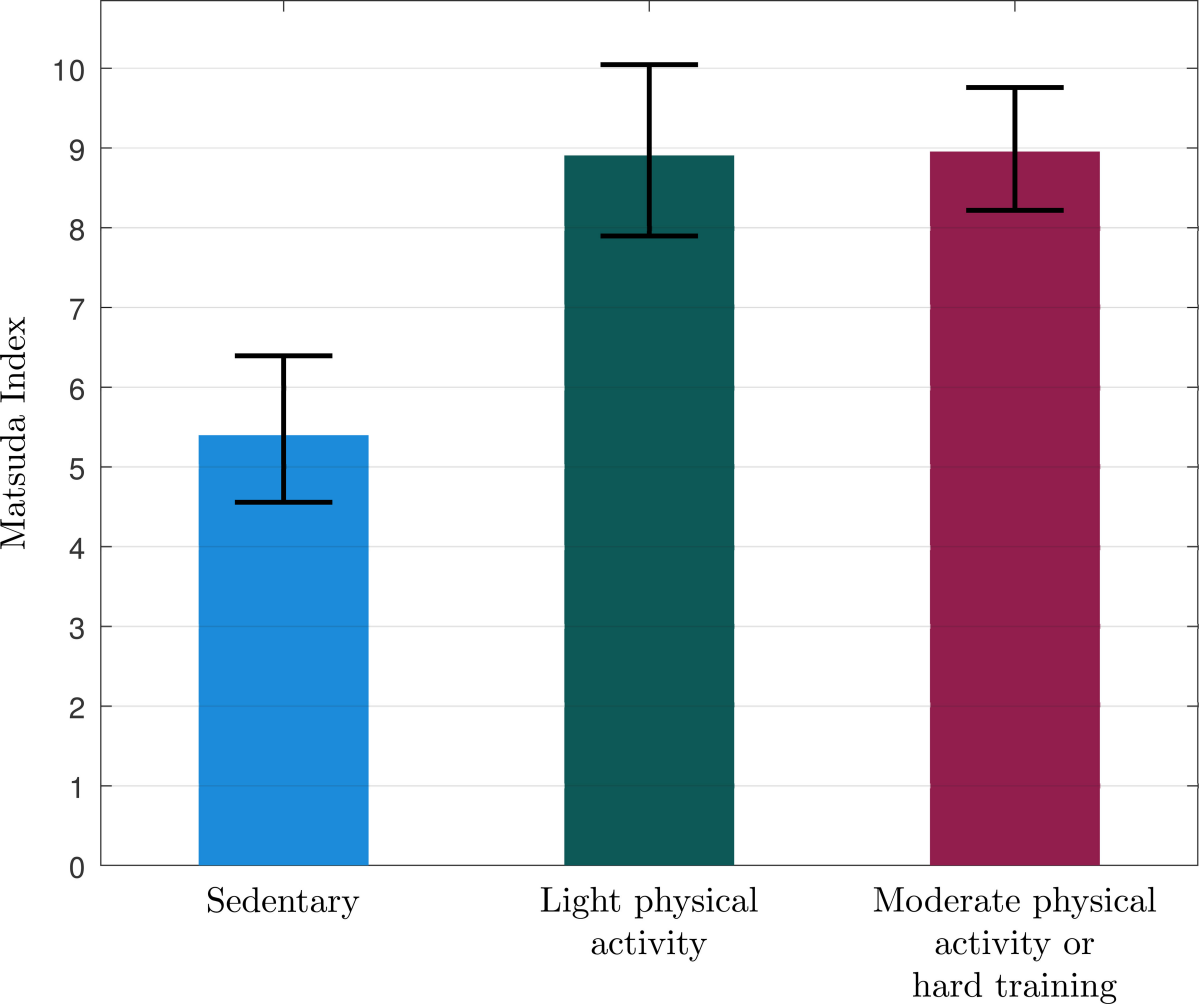
Mean (95% CI) of Matsuda Index in the burnout subjects reporting that they are sedentary (n=11), practice some light physical activity (n=13), or regular physical activity (n=14).

**Figure 4:**
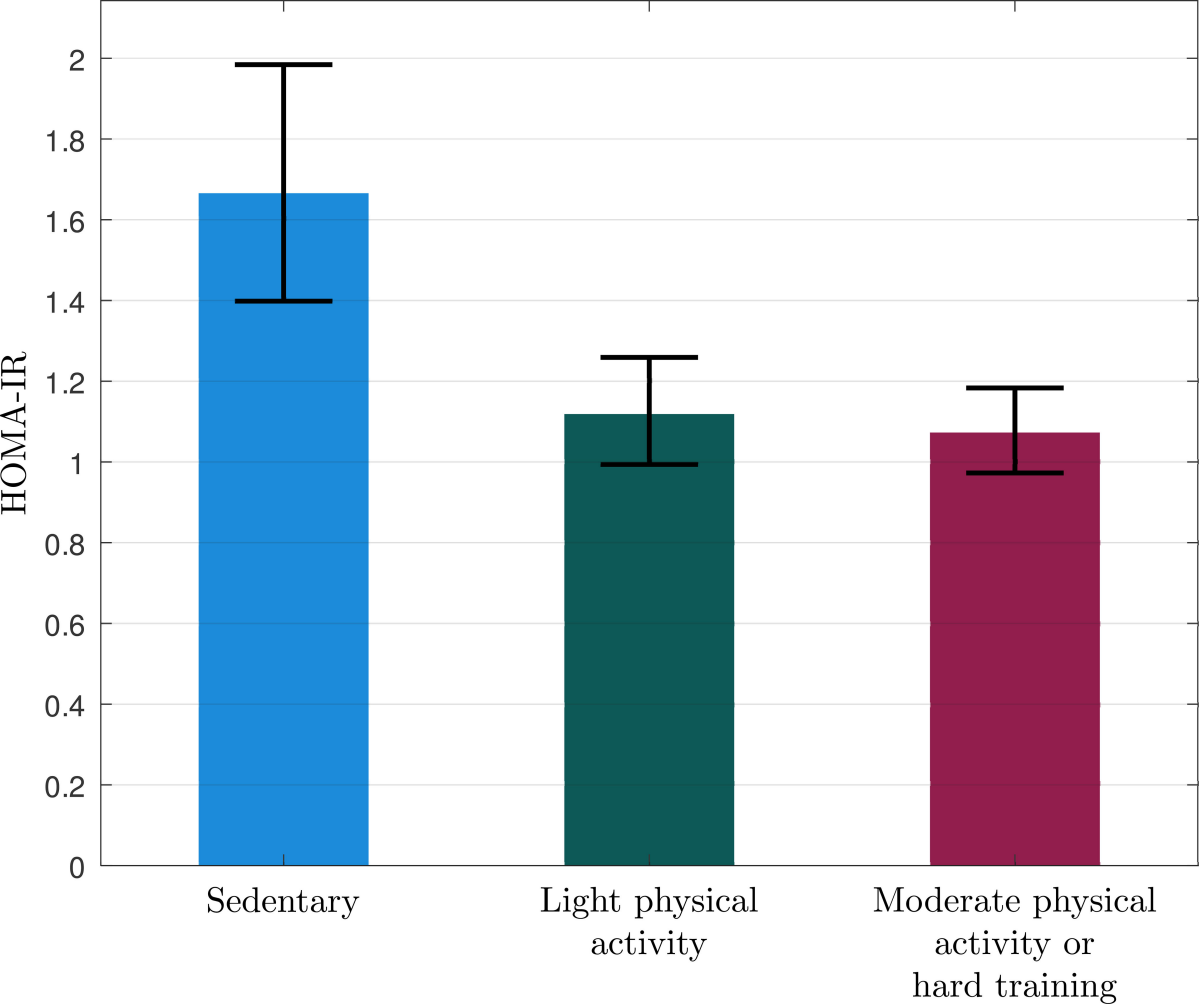
Geometric mean (95% CI) of HOMA-IR in the burnout subjects reporting that they are sedentary (n=11), practice some light physical activity (n=13), or regular physical activity (n=14).

**Figures 1**–**4** show that the two groups who were physically active exhibited very similar values. Therefore, in further statistical analysis, these groups were merged and managed as one group and named Active. Statistical analyses were performed comparing sedentary and active burnout cases. There were no significant differences in age (39 vs 43 years, p=0.156), proportion of males/females (36 vs 33% men, p=0.858), BMI (23.2 vs. 22.0kg/m^2^, p=0.441) or severity of burnout symptoms (median 5.7 vs. 5.5, p=0.373) or depression (median 7 vs. 7, p=0.323) between sedentary and active burnout cases. Fasting glucose or HbA1c did not differ between sedentary and active burnout cases (5.4 vs 5.2, p=0.197 and 31 vs 32, p=0.229). Glucose levels during OGTT (see **Figure 1**, **Table 2**) did not differ significantly between sedentary and active burnout cases (F = 1.596, p=0.215). AUC glucose did not differ significantly between sedentary and active (**Table 2**).

**Table 2:** Geometric mean (95% CI) glucose and insulin levels during oral glucose tolerance test (OGTT), AUC glucose, AUC insulin, Matsuda index, and HOMA-IR in sedentary and physically active burnout cases.

Glucose and insulin levels	Sedentary	Active	t	p value	Hedges’ g
	n=11	n=27			
Glucose 0 min	5.3 (5.1-5.6)	5.1 (4.9-5.3)	1.506	0.141	0.089
30 min	8.1 (7.3-9.0)	7.2 (6.6-7.8)	1.615	0.115	0.202
60 min	6.8 (5.7-8.2)	6.1 (5.5-6.8)	1.248	0.220	0.274
90 min	5.8 (4.9-6.9)	5.4 (4.8-6.0)	0.810	0.423	0.280
120 min	5.6 (4.9-6.3)	5.3 (4.8-5.9)	0.453	0.653	0.249
Insulin 0 min	7.0 (4.9-10)	4.8 (4.2-5.5)	2.598	**0.014**	0.410
30 min	58 (43-79)	38 (32-45)	2.799	**0.008**	0.445
60 min	59 (38-92)	35 (29-42)	2.853	**0.007**	0.542
90 min	42 (26-69)	25 (20-31)	2.440	**0.020**	0.603
120 min	43 (26-71)	23 (18-29)	2.783	**0.009**	0.643
AUC glucose	791 (713-877)	740 (706-777)	1.313	0.198	0.198
AUC insulin	5791 (4229-7928)	4032 (3615-4496)	3.531	**0.001**	0.416
Matsuda Index	6.18 (3.93-8.44)	9.48 (8.18-10.8)	2.816	**0.008**	3.321
HOMA-IR	1.67 (1.13-2.46)	1.09 (0.94-1.28)	2.605	**0.013**	0.460

Bold p-value indicates significant difference, p<0.05.

Insulin levels during the OGTT are reported in **Figure 2**, **Table 2**. The sedentary burnout cases exhibited significantly higher insulin levels during OGTT than the patients who were physically active (F = 11.922, p=0.001). AUC insulin was significantly higher in the sedentary participants than the active (**Table 2**), even after adjusting for age, BMI, and sex (**Table 3**). Matsuda Index was significantly lower, and HOMA-IR was significantly higher in sedentary than in physically active subjects (**Table 2**), even after adjusting for age, BMI, and sex (**Table 3**). Physical activity level (sedentary vs. active) explained 14-21% of the variation of insulin sensitivity (**Table 3**). There were no significant differences in insulin levels within active burnout cases, thus cases reporting light physical activity and moderate to hard activity exhibited similar levels (data not shown).

**Table 3:** Regression analyses predicting insulin sensitivity (Model 1: AUC insulin; Model 2: Matsuda Index; Model 3: HOMA-IR) in 13 male and 25 female burnout cases who reported that they were sedentary (n=11) or physically active (n=27).

Regression Models	B	95% CI	Beta	t	R^2^-change	p
Model 1: AUC insulin						
Age	-0.002	-0.021−0.016	-0.041	-0.260	0.017	0.796
BMI	0.021	-0.027−0.069	0.146	0.883	0.023	0.384
Sex	-0.186	-0.505−0.134	-0.192	-1.184	0.045	0.245
Physical activity	-0.491	-0.811−-0.170	-0.486	-3.119	0.213	**0.004**
Model 2: Matsuda Index						
Age	-0.031	-0.179−0.117	-0.071	-0.424	0.001	0.674
BMI	-0.060	-0.449−0.329	-0.056	-0.315	0.023	0.755
Sex	-0.423	-3,010−2.164	-0.058	-0.333	0.000	0.742
Physical activity	3.367	0.769−5.964	0.440	2.640	0.174	**0.013**
Model 3: HOMA-IR						
Age	0.004	-0.015−0.023	0.065	0.410	0.001	0.684
BMI	0.018	-0.032−0.069	0.123	0.742	0.083	0.464
Sex	0.262	-0.072−0.595	0.260	1.598	0.046	0.120
Physical activity	-0.413	-0.749−-0.077	-0.392	-2.502	0.139	**0.017**

Sex: 1=Female vs. 2=Male. Physical activity: 1=Sedentary vs. 2=Active.

Bold p-value indicates significant contribution, p>0.05.

## 4 Discussion

Analyzing burnout cases with varying levels of physical activity over the past year, we observed a significant difference in insulin sensitivity and insulin levels during the OGTT between sedentary individuals and those who were physically active. Thus, a significant difference was observed between the sedentary patients and physically active patients in insulin levels during OGTT as well as in HOMA-IR (insulin resistance) and Matsuda Index (insulin sensitivity), regardless of physical activity level (light or moderate and hard physical activity). The relation between physical activity and insulin sensitivity is well known in healthy individuals as well as in individuals with type 2 diabetes (14, 15). It has been established that acute exercise - as well as regular exercise - causes substantial improvement in glucose metabolism and insulin sensitivity (25–28). The present study indicates that the expected relationship between physical activity and insulin sensitivity is seen even among burnout subjects. To our knowledge, the relation between physical activity level and glucose control has not been studied previously in burnout cases.

### 4.1 The association was independent of severity of symptoms

In a previous study (16) we found that the burnout cases with more severe burnout symptoms exhibited significantly higher levels of both glucose and insulin levels during the OGTT compared to burnout cases reporting lower severity of symptoms. In addition, the group of burnout cases who reported symptoms of depression exhibited higher insulin levels during OGTT compared to the burnout cases without depressive symptoms. It could be speculated that the relation between physical activity and glucose control may be disturbed in this patient group due to previous extensive stress exposure and that the symptoms of burnout and depression might have a greater impact on insulin sensitivity than the level of physical activity. However, the burnout cases who reported severe burnout and depression reported equal level of self-reported physical activity. Accordingly, there was no statistical difference between the sedentary and physically active in scores of burnout or depression. Thus, in this study, reported physical activity seems to be independent of the severity of symptoms in burnout subjects. However, previously we have shown that better compliance to physical activity recommendation among patients with burnout was shown to be related to symptom reduction of both burnout and depression (29).

### 4.2 Clinical relevance

Prospective studies have shown that burnout increase risk of incident of type 2 diabetes by twofold or threefold (5, 9). The sedentary burnout cases in the present study show signs towards pre-diabetes, as indicated by elevated insulin levels, higher HOMA-IR, and lower Matsuda index. This adds to the rationale for promoting physical activity in burnout patients. There is evidence for good effect of physical activity on reducing exhaustion in intervention studies (30) This study points towards additional reasons for the importance of physical activity in burnout patients, thus, in preventing type 2 diabetes.

### 4.3 Methodological considerations

Firstly, it should be mentioned that the small study sample is to be considered as a limitation, but even with the limited number of participants the results were significant. The original plan was to recruit additional patients, but many individuals with burnout are simply too exhausted to participate in research studies that require efforts such as the OGTT challenge. One methodological consideration to be raised is the measurement of physical activity since a simple self-rated measure has been used. The Saltin-Grimby Physical Activity Level Scale (31) to assess physical activity level among the subjects during the past year and related their responses to glucose and insulin metabolism. This is a single item question with four response options. The first level corresponds to a sedentary lifestyle, while levels 2–4 represent graded increases in activity level from light to strenuous exercise training. This measure has been shown to be useful as screening measure and the different level of physical activity was shown to be clearly related to different cardiometabolic measures in both women and men (24). An optional methodology to investigate the research question would be to use for example pedometers to objectively measure physical activity level (32). Since 3 participants were tobacco users it should be mentioned that smoking and snuff (33, 34) may increase the risk of type 2 diabetes and thus may influence the results. Anti-depressant medication may also potentially affect insulin sensitivity. However, there were equal proportions of tobacco users and antidepressant users, respectively, between sedentary and active (shown in **Table 1**) and *post hoc* analysis performed excluding the three tobacco users or antidepressant users did not change the results (data not shown). The study individuals were selected to have normal BMI and to be otherwise healthy, thus we do not know if the results would be valid for individuals with burnout who were overweight, underweight, or having other health issues.

## 5 Conclusion

The observed higher insulin levels/lower insulin sensitivity in the sedentary burnout cases, compared to the physically active burnout cases, may indicate higher diabetes risk in these individuals and points at an additional reason for including physical activity in the treatment for this patient group. Future studies could investigate intensity and duration of physical activity needed to positively change insulin sensitivity in sedentary individuals with burnout.

## Data Availability

The raw data supporting the conclusions of this article will be made available by the authors, without undue reservation.
